# Genetic Diversity and Association Mapping for Agromorphological and Grain Quality Traits of a Structured Collection of Durum Wheat Landraces Including subsp. *durum*, *turgidum* and *diccocon*

**DOI:** 10.1371/journal.pone.0166577

**Published:** 2016-11-15

**Authors:** Patricia Giraldo, Conxita Royo, Mirvana González, Jose M. Carrillo, Magdalena Ruiz

**Affiliations:** 1 Department of Biotechnology-Plant Biology, School of Agricultural Engineering, Technical University of Madrid, Madrid, Spain; 2 Field Crops Program, Institute for Food and Agricultural Research and Technology, Lleida, Spain; 3 Laboratory of Bioinformatics and Biofonotics, Engineering Institute, Autonomous University of Baja California, Mexicali, México; 4 Plant Genetic Resources Centre, National Institute for Agricultural and Food Research and Technology, Alcalá de Henares, Spain; Università Politecnica delle Marche, ITALY

## Abstract

Association mapping was performed for 18 agromorphological and grain quality traits in a set of 183 Spanish landraces, including subspecies *durum*, *turgidum* and *dicoccon*, genotyped with 749 DArT (Diversity Array Technology) markers. Large genetic and phenotypic variability was detected, being the level of diversity among the chromosomes and genomes heterogeneous, and sometimes complementary, among subspecies. Overall, 356 were monomorphic in at least one subspecies, mainly in *dicoccon*, and some of them coincidental between subspecies, especially between *turgidum* and *dicoccon*. Several of those fixed markers were associated to plant responses to environmental stresses or linked to genes subjected to selection during tetraploid wheat domestication process. A total of 85 stable MTAs (marker–trait associations) have been identified for the agromorphological and quality parameters, some of them common among subspecies and others subspecies-specific. For all the traits, we have found MTAs explaining more than 10% of the phenotypic variation in any of the three subspecies. The number of MTAs on the B genome exceeded that on the A genome in subsp. *durum*, equalled in *turgidum* and was below in *dicoccon*. The validation of several adaptive and quality trait MTAs by combining the association mapping with an analysis of the signature of selection, identifying the putative gene function of the marker, or by coincidences with previous reports, showed that our approach was successful for the detection of MTAs and the high potential of the collection to identify marker–trait associations. Novel MTAs not previously reported, some of them subspecies specific, have been described and provide new information about the genetic control of complex traits.

## Introduction

Durum wheat (*Triticum turgidum* L. subsp. *durum*) is an important food crop grown on about 13 million hectares worldwide. This wheat subspecies is mostly used for pasta manufacturing, but it is also the raw material for producing other traditional goods such as flat breads, couscous and bulgur. In the Mediterranean basin, it is a crop of strategic importance with about 60% of the world durum-growing area. In this region, durum wheat is grown in a range of climatic zones varying from warm and dry to cool and wet [1]. However, the massive introduction of homogeneous and more productive modern cultivars have contributed to greatly reduce its genetic diversity in comparison to the landraces widely grown until the late 1960s. An autochthonous wheat landrace is defined as a traditional variety adapted to local conditions with a high capacity to tolerate biotic and abiotic stresses [2] and represents an intermediate stage in domestication between wild wheat and elite cultivars. In fact, allelic variation of genes originally found in the wild, but gradually lost through domestication and breeding, have been recovered only by going back to landraces [3]. So, wheat landrace collections contain wider genetic diversity than most breeding programmes and constitute an easily transferable and valuable source of genetic variation for agronomical, morphological, adaptive and quality traits.

Discovery of new markers associated with key traits in landrace collections has important implications for durum wheat breeding, particularly after validation of significant markers associated with complex traits of economic importance. Genomics approaches enable the identification and selection of chromosome regions harbouring genes/QTLs (Quantitative Trait Loci) controlling agronomic traits in crops [4]. Among such approaches, association mapping is increasingly being adopted as a complementary method to traditional bi-parental linkage mapping to identify genotype–phenotype associations in wheat e.g. [5–8]. The underlying principle of this approach is that linkage disequilibrium (LD) tends to be maintained over many generations between loci that are genetically linked to one another. High LD is expected to be observed between loci in tight linkage, as recombination events since the mutation should have eliminated LD between loci that are not in close distance [9]. The two major advantages of association mapping over standard genetic mapping based on populations of bi-parental crosses are, firstly, that a much larger and more representative gene pool can be surveyed, and, secondly, provides broader genomic region/allelic coverage without the need to develop bi-parental mapping populations. Another advantage is the much finer mapping resolution, resulting in small confidence intervals of the detected loci compared to classical mapping, where the identified loci need to become fine-mapped [10]. It is important, however, separate the LD due to physical linkage from the LD due to population structure which can be caused by selection, genetic drift and species-dependent characters such as the mating system [11]. The choice of appropriate germplasm to maximize the number of historical recombination and mutation events (and thus reduce LD) within and around the gene of interest is critical for the success of association analysis. One of the methods to obtain most of the phenotypes is to construct a core subset, which includes in the sample as much variability present in the whole collection as possible with a minimum of redundancies [12].

Mediterranean *durum* landraces represent a particularly important group of genetic resources because of their extensive genetic variability [13, 14]. A diversification of the durum wheat species (*Triticum turgidum* L.) into different subspecies occurred in several territories. This was the case in Spain, whose durum wheat landraces have been classified into three main inter-fertile subspecies, *dicoccon*, *turgidum*, and *durum*, which all share the same AABB genomic constitution. Each subspecies had a different eco-geographical distribution and end use [15, 16]. Subspecies *dicoccon* (emmers), a hulled wheat for animal feed and bread making, represents a primitive stage in crop evolution, and was gradually replaced by more advanced free-threshing types of subsp. *durum* and *turgidum* (rivets or cone wheat), which evolved from subsp. *dicoccon* [17]. Cultivation of emmers was restricted to mountainous regions. In contrast, the subsp. *durum* were the most widely grown and adapted to dry environments; rivets were less common and were grown in colder areas than *durum*. Although both subspecies were consumed as pasta and semolina products, landraces of *turgidum* had, in general, lower quality and higher tillering rate than the *durum* types [15]. Several studies have shown that all these landraces have a great variability relative to other germplasm collections [13, 16, 18–21].

In a previous study, a durum wheat core subset of Spanish landraces comprising accessions of the three subspecies *durum*, *turgidum* and *diccocon* was constructed [16]. Moreover, we evaluated the diversity and genetic structure of this core subset using several marker systems and correlated the diversity and agromorphological traits with geographic and climatic features.

In this study, association mapping was performed for 18 agromorphological and grain quality traits in the core subset genotyped with DArT (Diversity Array Technology) markers. These data were used to: (i) investigate the genetic and phenotypic diversity, (ii) identify marker–trait associations (MTAs); and (iii) test the contribution of each subspecies to general diversity of the whole collection and their potential as a source of genetic variation for wheat improvement.

## Material and Methods

### Germplasm, genotyping and phenotypic evaluation

A total of 183 wheat genotypes of three subspecies: 132 of *T*. *turgidum* subsp. *durum*, 38 of *T*. *turgidum* subsp. *turgidum*, and 13 of *T*. *turgidum* subsp. *dicoccon*, were used for this study [16]. The collection had been previously genotyped with DArT arrays, being polymorphic for 749 markers [16]. The correspondence between DArT markers and *Brachypodium* genomic sequences was established from previous information [22]. The genomic sequences related to DArT markers were obtained from Diversity Array Technology (http://www.diversityarrays.com/dart-map-sequences). In both cases gene ontology was addressed in PlantGBD database (http://www.plantgdb.org/BdGDB/cgi-bin/search.pl).

Phenotyping was conducted under field conditions in three diverse Spanish locations: Lleida in the North (N), Alcala de Henares in the Centre (C), and Jerez in the South (S) of the country. No specific permissions were required for field studies because they were carried out in fields from public research centers. The field studies did not involve endangered or protected species. Experiments were carried out over the season November 2006 to June 2007, and in the South also in 2007–2008 (S08). The description of the four testing environments (combinations of location and year) is shown in S1 Table. Field experiments consisted of non-replicated plots of 1.5 m^2^ in C, and 0.6 m^2^ in N and S arranged according to a modified augmented design with four checks common to all experiments. This design was chosen because it allowed testing such a large number of entries saving land and management costs, having the additional advantages of being independent of the homogeneity of error variance and of the model of response at each environment [23]. The accessions were evaluated for six qualitative agromorphological traits in C (plant growth **PG**, spike density **SD**, glume hairiness **GH**, glume colour **GC**, awn colour **AC**, and seed colour **SC**), six quantitative agromorphological traits (days to heading **DH**, plant height **PH**, number of spikelets per spike **SS**, days to maturity **DM**, carbon isotope discrimination **Δ**^**13**^**C** and spike length **SL**) and six grain quality traits (protein content **PC,** gluten strength **GS**, vitreousness **V**, yellow colour index b **YI**, thousand-kernel weight **TKW** and test weight **TW**), assessed in different environments. Agromorphological traits were recorded according to IBPGR [24] from five representative plants of each accession. Heading and physiological maturity dates were recorded when more than 50% of the main spikes within a plot had reached Zadoks stage 55 and 87, respectively [25] and the days from sowing to these stages were calculated for each plot. A sample of about 2 g of matured kernels from each plot was finely ground (to pass a 0.5-mm sieve) for carbon isotope analysis. The ^13^C to ^12^C ratios were determined by mass spectrometry at Isotope Services Inc., Los Alamos, NM. Stable carbon isotope composition was expressed as δ13C values [26], where δ^13^C (‰) = [(R_grain_/R_standard_)-1] × 1000, and R was the ^13^C to ^12^C ratio. Secondary standards of graphite, sucrose, and polyethylene foil calibrated against Pee Dee belemnite (PDB) carbonate were used for comparison. The accuracy of the δ^13^C measurements was ±0.1‰. Carbon isotope discrimination was further computed as Δ^13^C = (δ_a_−δ_s_)/(1+δ_s_), where δ_a_ and δ_s_ refer to δ^13^C of air and plant, respectively [27]. On the PDB scale, free atmospheric CO_2_ has a current deviation, δ_a_, of approximately –8‰ [26]. Grain protein content (%) was determined by a near infrared spectroscope (NIT, Infratec^®^ 1,241-grain analyzer, Foss Tecator AB, Sweden) calibrated against the standard Kjeldahl method. Gluten strength was determined on 1 g of whole grain flour samples by the SDS (sodium dodecyl sulphate) sedimentation test, using the method of Axford et al. [28] as modified by CIMMYT, using stoppered 25 ml graduated cylinders. Vitreousness was determined by cutting transversally with a scalpel 100 kernels taken at random on each sample and identifying those not fully vitreous according to the appearance of the sectional areas of the endosperm. Yellow colour index was estimated on whole grain flour by means of a reflectance colorimeter (CR-400, Konica-Minolta) equipped with a filter tri-stimulate system. Thousand kernel weight was estimated as the mean weight of three sets of 100 grains per plot. Test weight was determined by the GAC2100 Dickey-John equipment, and expressed as kg hl^-1^.

### Data analysis

Phenotypic data were fitted to a linear mixed model with the check cultivars as fixed effects, and the row number, column number and accession as random effects [29]. Restricted Maximum Likelihood (REML) was used to estimate the variance components and to produce the Best Linear Unbiased Predictors (BLUPs) for the phenotypic data of each accession within each environment, achieved using the MIXED procedure of the SAS-STAT statistical package [30].

Gene diversity (*H*) within the whole collection and within subspecies were calculated according to Nei [31]. The relative loss of genetic diversity in the whole collection *vs*. the subspecies was measured according to Vigouroux et al. [32], using the parameter *ΔGD* = 1-(*H*_subsp_/ *H*_wc_), where *H*_subsp_ and *H*_wc_ are genetic diversity in the subspecies and the whole collection, respectively.

The Linkage Disequilibrium was assessed between the 329 DArTs that could be mapped on the durum wheat consensus linkage map constructed by Marone et al. [33]. The LD (allele frequency correlation, r) estimates between marker pairs were obtained using the TASSEL v5 software [34]. The significance of the pairwise LD (P values) was computed using 1,000 permutations. The intrachromosomal r values were plotted against the genetic distance, to see how the LD decay occurs. According to Breseghello and Sorrells [5], unlinked- *R*^*2*^ estimates were square root transformed to approximate a normally distributed random variable; then the parametric 95th percentile of that distribution was taken as a population-specific critical value of *R*^*2*^, beyond which LD was likely to be caused by genetic linkage. The intersection of the loess curve fit to syntenic *R*^*2*^ with this baseline was considered as the estimate of the extent of LD in the chromosome [35].

Overall, 532 DArTs were selected for association analyses, for having less than 5% of missing data. TASSEL v5, was used to perform association mapping analysis using the General Linear Model (GLM) with population structure (Q model). The genetic structure had been previously investigated using the software STRUCTURE [16]. Random variables association values and Bonferroni correction were applied. Marker alleles significantly associated in Tassel analysis (*P* < 0.0005) and confirmed with χ^2^ test (*P* < 0.01) for qualitative variables, or with simple linear regression analysis (*P* < 0.05 in more than one environment or subspecies) for quantitative variables, were declared significantly associated with the trait evaluated.

Analyses of variance (ANOVA) was performed to determine the effects of subspecies and environment on the phenotypic variables. Relationships between variables were examined using Pearson and Spearman correlation coefficients for normal and no normal variables, respectively. The analysis were performed with the statistical software R 3.2.3 [36] and Infostat [37] statistical packages.

## Results

### Phenotypic evaluation

Table 1 shows the frequency of the qualitative agromorphological traits within each subspecies. Data analyses with χ^2^ test detected significant differences (*P*<0.05) among the three subspecies, except for GC. In general, subsp. *durum* had dense spikes, with black awns at the base and white seeds. Conversely, subsp. *dicoccon* had less dense hairless spikes with red seeds. None *dicoccon* had erect habit.

**Table 1:** Relative frequency (%) of the qualitative agromorphological traits within the subsp. *durum*, *turgidum* and *dicoccon*.

Trait	Class	Subspecies		
		*durum*	*turgidum*	*dicoccon*
Plant growth	Prostrate	8.3	36.8	30.8
	Intermediate	75.0	60.5	69.2
	Erect	16.7	2.6	0.0
Spike density	Lax	3.8	2.6	7.7
	Intermediate	29.6	63.2	84.6
	Dense	62.1	21.1	7.7
	Very dense	4.6	13.2	0.0
Glume hairiness	Hairless	58.3	57.9	100.0
	Low	10.6	5.3	0.0
	High	31.1	36.8	0.0
Glume colour	White	45.5	52.6	76.9
	Red to brown	34.1	39.5	23.1
	Black	20.5	7.9	0.0
Awn colour	White	9.1	34.2	76.9
	Black at the base	81.8	44.7	0.0
	Red to brown	9.1	21.1	23.1
Seed colour	White	80.3	42.1	0.0
	Red	19.7	57.9	100.0

The statistical parameters of the quantitative traits for each subspecies and environment are shown in Table 2. High phenotypic variability was detected across the three subspecies, but also across the different landraces within each subspecies. The ANOVA indicated that there were significant differences in all traits among subspecies (S2 Table). Landraces of *durum* were the earliest in heading and maturity, and showed the lowest SS number, and the shortest spikes. In contrast, *dicoccon* had the shortest heading to maturity period, and the largest spikes with the highest number of SS, while wheats of subsp. *turgidum* were the tallest. For grain quality traits, *dicoccon* showed the highest PC, and the lowest GS, YI and TW values. Landraces of subsp. *durum* had the highest YI while those of *turgidum* showed the lowest TKW. There were significant differences in all the traits among environments, except for PC (S2 Table). Estimates of the variances due to subspecies x environment interaction were significant only for DH, V, TKW and TW. For V, the interaction effect was larger than the subspecies and environment effects. Larger influence estimates of subspecies variation compared to environment and subspecies x environment interaction were determined for SS number, Δ^13^C, PC, GS, YI and TKW, but smaller than environment variation for DH, PH, DM and TW. For DH, the order from early to late was S<S08<N<C. Plant height and DM were lined up in the same order as DH for the environments included in the evaluation. Lower TW values were obtained in the C environment than in the N (Table 2). For each subspecies, S3 and S4 Tables show the correlation coefficients between environments for each trait and between traits for each environment, respectively.

**Table 2:** Statistical estimations of the quantitative agromorphological and grain quality traits for the subsp. *durum*, *turgidum* and *dicoccon* at each environment (N north, C centre and S south).

		Mean			S.D.			Minimum			Maximum		
Traits	Environment	*durum*	*turgidum*	*dicoccon*	*durum*	*turgidum*	*dicoccon*	*durum*	*turgidum*	*dicoccon*	*durum*	*turgidum*	*dicoccon*
Quantitative agromorphological traits													
Days to heading	C	172.48	177.73	181.02	3.24	2.49	2.90	166.67	173.00	174.33	182.00	184.00	183.33
Days to heading	N	158.61	162.51	164.98	3.04	2.60	3.41	151.82	158.83	160.66	169.03	167.36	169.61
Days to heading	S	130.54	133.27	137.31	4.65	6.50	3.41	106.14	112.09	131.65	146.36	141.91	143.89
Days to heading	S08	133.74	139.82	145.69	3.77	5.12	3.38	127.01	132.02	139.00	143.02	153.00	153.01
Plant height (cm)	C	122.51	133.27	117.17	9.27	8.38	5.62	94.58	115.92	107.25	146.58	149.92	127.25
Plant height (cm)	N	117.22	131.06	120.00	11.82	10.17	8.08	85.40	113.18	106.72	141.98	155.86	131.74
Plant height (cm)	S	103.83	112.60	105.78	9.67	7.44	8.91	76.83	95.91	88.27	128.96	124.30	121.19
Spikelets per spike (number)	C	22.42	24.98	26.13	1.71	6.46	3.65	18.65	18.90	20.90	29.06	61.40	31.90
Spikelets per spike (number)	N	20.69	23.30	24.75	1.29	1.94	3.29	17.47	19.81	18.96	24.91	26.81	29.06
Spikelets per spike (number)	S	21.55	23.86	24.64	2.24	2.93	2.35	16.32	17.91	21.33	29.13	32.47	28.21
Days to maturity	C	213.23	215.20	216.33	3.76	1.53	3.34	200.92	213.92	214.33	219.67	218.33	223.33
Days to maturity	N	193.17	196.66	195.52	2.18	1.96	2.95	188.97	192.55	190.33	201.89	200.42	200.35
Δ^13^C	C	17.07	16.74	16.68	0.51	0.66	0.41	15.89	15.54	16.18	18.18	19.48	17.50
Δ^13^C	N	17.25	17.03	17.14	0.20	0.18	0.16	16.60	16.65	16.92	17.70	17.35	17.48
Spike length (cm)	C	95.37	107.88	125.56	11.43	10.82	20.57	71.96	77.79	96.79	146.63	129.63	162.79
Grain quality traits													
Protein content (%)	C	16.56	17.45	19.08	2.65	2.74	1.53	10.24	11.61	17.00	20.38	21.67	22.33
Protein content (%)	N	16.81	16.90	17.75	0.90	1.28	1.09	14.84	14.38	16.18	19.27	19.01	19.38
Gluten strength (mm)	C	6.40	6.31	5.50	2.08	1.48	1.47	2.56	3.35	3.77	11.35	9.27	8.77
Gluten strength (mm)	N	6.85	6.73	5.77	1.67	1.27	0.87	3.83	3.94	4.32	11.00	9.23	7.81
Vitreousness (%)	C	97.49	85.29	86.75	3.91	14.34	10.94	74.71	35.13	65.13	101.71	99.13	99.13
Vitreousness (%)	N	89.22	92.52	90.17	11.35	3.16	5.47	24.66	84.92	72.60	98.75	99.47	93.66
Yellow Index	C	14.24	13.21	11.79	0.94	0.87	0.51	11.89	11.46	10.72	17.28	16.45	12.59
Yellow Index	N	15.08	14.39	12.81	0.91	0.88	0.46	12.88	13.02	11.83	17.48	17.44	13.57
Thousand kernel weight (g)	C	53.63	50.76	62.02	8.25	5.85	8.81	34.63	38.63	38.00	74.29	63.63	72.00
Thousand kernel weight (g)	N	53.38	48.07	50.46	5.32	5.23	9.76	41.02	39.55	31.99	67.51	62.41	61.74
Test weight (kg/hl)	C	77.62	77.91	76.73	1.86	1.49	2.03	71.73	74.73	73.29	81.66	81.62	80.62
Test weight (kg/hl)	N	80.91	79.61	79.02	1.97	1.90	1.82	73.79	74.09	76.61	84.93	83.07	80.66

All experiments were conducted in 2007 except in the south in 2008 as indicated (S08).

### Genetic diversity and linkage disequilibrium

To understand the overall genetic diversity among the whole collection and the three subspecies, the genetic diversity (*H*) and the relative loss of diversity (*ΔGD*) and chromosome were evaluated (Table 3). The results obtained with DArT markers indicated that *H* values were 0.36 for the whole collection, and 0.32, 0.28 and 0.16 for subsp. *durum*, *turgidum* and *dicoccon*, respectively. A diversity of 12, 22 and 56% remains in the collection if the subsp. *durum*, *turgidum* or *dicoccon*, respectively, are removed from the whole collection. Genetic variability also differed on its distribution among chromosomes among subspecies.

**Table 3:** DArT markers index of diversity (*H*) and relative loss of diversity (*ΔGD*) in the whole collection vs. the subspecies.

Chr.	*H*				*ΔGD*		
	Whole collection	*durum*	*turgidum*	*dicoccon*	*durum*	*turgidum*	*dicoccon*
1A	0.41	0.39	0.29	0.22	0.06	0.30	0.47
1B	0.31	0.28	0.25	0.12	0.11	0.18	0.62
2A	0.37	0.36	0.23	0.19	0.05	0.38	0.49
2B	0.35	0.33	0.19	0.12	0.06	0.46	0.65
3A	0.38	0.34	0.38	0.15	0.11	0.02	0.61
3B	0.35	0.33	0.26	0.16	0.06	0.26	0.55
4A	0.39	0.34	0.30	0.14	0.13	0.23	0.63
4B	0.33	0.20	0.33	0.12	0.39	0.01	0.64
5A	0.29	0.19	0.27	0.18	0.34	0.09	0.37
5B	0.36	0.34	0.28	0.19	0.07	0.24	0.48
6A	0.41	0.36	0.33	0.16	0.12	0.19	0.62
6B	0.39	0.37	0.28	0.13	0.04	0.27	0.66
7A	0.33	0.28	0.30	0.16	0.15	0.08	0.53
7B	0.35	0.32	0.23	0.17	0.07	0.34	0.50
Genome A	0.37	0.32	0.30	0.17	0.13	0.19	0.54
Genome B	0.35	0.31	0.26	0.14	0.11	0.26	0.59
Total	0.36	0.32	0.28	0.16	0.12	0.22	0.56

A total of 11, 80 and 265 DArT markers were monomorphic in the *durum*, *turgidum* and *dicoccon* subspecies, respectively (S5 Table). Fifty-one markers were simultaneously fixed in *turgidum* and *dicoccon*, while *durum* only matched in three and four monomorphic markers with *turgidum* and *dicoccon*, respectively.

Linkage disequilibrium analysis was performed for the whole collection. Pairwise LD was estimated using the squared allele frequency correlations (*R*^*2*^). The LD pattern in the whole collection was assessed based on the 108,241 pairwise combinations of 329 DArTs. Pairwise *R*^*2*^ estimates among varied from 2.75e-8 to 1, with a median of 0.05492. The plots of the LD estimates (r) as a function of genetic distance in centiMorgans (cM) showed the decay of the LD with genetic distance (S1 Fig). The 95 percentile of the LD estimate distribution was used as the critical threshold to better discriminate the LD that was most likely due to physical linkage, *R*^*2*^ = 0.0106. The intersection of the loess curve fit to syntenic *R*^*2*^ with this baseline was considered as the estimate of the extent of LD in the chromosome = 7.3cM.

### Association analysis

#### Association with qualitative agromorphological traits

DArT markers significantly associated with the qualitative agromorphological traits were identified for each subspecies within each environment (Table 4). Markers wPt-6509(3A) and wPt-1151(3BL) were significantly associated with **PG** in *durum* and *dicoccon*. Both markers were significantly correlated in the three subspecies (*P*<0.01). For **SD**, three markers showed association in *durum* and one, in *turgidum*. Three out of them were monomorphic in the subsp. *turgidum* or *dicoccon* (S5 Table). Marker wPt-4676 (1AS) was associated with **GH** in *durum* and *turgidum* (Table 4), with a *R*^2^ value of 0.48. This marker was monomorphic in the *dicoccon*, all of them with hairless glumes (Table 1). Two markers on chromosome 1BS, wPt-2395 and wPt-8267, were identified as MTAs for **GC** with a *R*^*2*^ value of 0.32 and 0.13, respectively. Marker wPt-2395 was associated with white glumes in the three subspecies, and wPt-8267 in *durum* and *dicoccon*. Marker wPt-2395(1BS) was also associated with white and red awns in the subsp. *durum* and *turgidum*. These two markers on chromosome 1BS were correlated in *durum* and *dicoccon* (*r* = 1) but not in *turgidum* (*r* = -0.10). Two markers on chromosome 3BL and two on group 6 showed association with white and red seed in *durum* and *turgidum*, respectively. Markers rPt-0996(3BL) and wPt-1830(6BS) were monomorphic in *dicoccon*, all of them with red seeds (Table 1 and S5 Table).

**Table 4:** Markers significantly associated with the qualitative agromorphological traits for the whole collection confirmed in the subsp. *durum*, *turgidum* and *dicoccon* with χ^2^ test.

Trait	marker	Chr.	*durum*	*turgidum*	*dicoccon*
Plant growth	wPt-6509	3A	11.01**		13.00**
	wPt-1151	3BL	11.01**		13.00**
Spike density	wPt-1374	1BS	31.76**		
	wPt-6825	5AL	33.29**		
	wPt-9094	5AL		11.81**	
	wPt-5462	7BL	27.34**		
Glume hairiness	wPt-4676	1AS	104.81**	18.04**	
Glume colour	wPt-2395	1BS	68.35**	20.43**	7.88**
	wPt-8267	1BS	69.01**		8.00**
Awn colour	wPt-2395	1BS	26.29**	8.24**	
Seed colour	rPt-0996	3BL	42.44**		
	wPt-0665	3BL	37.25**		
	wPt-7906	6AS		15.01**	
	wPt-1830	6BS		9.83**	

*, ** significant at *P*<0.05 and *P*<0.01, respectively

#### Association with quantitative agromorphological and grain quality traits

The associations detected with Tassel were confirmed by linear regression analysis for each subspecies within each environment. Only those markers confirmed in more than one environment or subspecies were considered significantly associated with the trait evaluated (Table 5). Ten markers showed significant association with **DH**, most of them across more than two environments. Only two matched in more than one subspecies. In subsp. *durum*, the wPt-6498(7BL) was confirmed in the four environments. In the *dicoccon* landraces analysed, marker tPt-2163(2A) showed the same genotype as wPt-1330(7BL), and wPt-2858(2AL) as wPt-7572(6A); four markers were significantly correlated in the three environments (*P* < 0.01). Four markers showed significant association with **PH** and two coincided in the subsp. *durum* and *turgidum*. One of them, wPt-4419(5A), explained 9–13% of the phenotypic variation in centre and northern environments in those subspecies. None MTA was found in *dicoccon* and only one was confirmed in the south, where plants were significantly shorter. The markers tPt-5080(1BS), wPt-4419(5A) and wPt-8981(7BL) were monomorphic in that subspecies (S5 Table). Fourteen markers showed significant association with the number of **SS**. Twelve of these MTAs were identified in the subsp. *dicoccon* at the three environments tested; four markers were located on the group 7 of chromosomes, three on group 1 and three on group 3. Among these markers, wPt-4120(7B) was monomorphic in *durum*, tPt-2163(2AL) in *turgidum* and wPt-1330(7BL) in both subspecies (S5 Table). Nine out of the 14 markers identified were completely correlated in *dicoccon* (*r* = 1 or -1). The wPt-8168(1BL) and wPt-2202(3AL) showed significant association in two and the three subspecies, respectively, and at more than one environment. The marker wPt-1163 (7AS), significantly associated in *turgidum* at the three environments, was monomorphic in *dicoccon* (S5 Table). Only the marker wPt-7426 (6BS) was confirmed for **MD**, being associated in the subsp. *turgidum* at the two environments evaluated. This marker was monomorphic in landraces of subsp. *dicoccon* and *durum* (S5 Table). Allelic variation of marker wPt-0696(6BL) was associated with lower **Δ**^**13**^**C** values in subsp. *turgidum* at both environments. Six markers showed association with the **SL**, two of them were located on chromosome 1BS and two on 3A. The markers tPt-6376(3A) and wPt-2202(3AL) concurred in the subsp. *durum* and *turgidum*, and *durum* and *dicoccon*, respectively. The two significant markers in *dicoccon* were completely correlated (*r* = -1). The wPt-1374 (1BS) associated with larger spikes in landraces of *durum* was monomorphic in the subsp. *turgidum*, and the wPt-1330 (7BL) associated with larger spikes in *dicoccon* was monomorphic in *turgidum* and *durum* landraces (S5 Table).

**Table 5:** Markers significantly associated with the quantitative agromorphological and the grain quality traits for the whole collection confirmed in the subsp. *durum*, t*urgidum* and *dicoccon* by lineal regression analyses.

Trait	Marker	chr.	*R*^*2*^ subsp. *durum*				*R*^*2*^ subsp. *turgidum*				*R*^*2*^ subsp. *dicoccon*			
			C	N	S	S08	C	N	S	S08	C	N	S	S08
			Quantitative agromorphological traits											
Days to heading	tPt-2163	2AL									0.45	0.61	0.35	
	wPt-2858	2AL					0.18		0.10		0.46	0.29	0.31	
	wPt-0489	2BL	0.12	0.06		0.04								
	wPt-3509	5AL					0.40	0.17		0.15				
	wPt-7572	6A									0.46	0.29	0.31	
	wPt-1890	6BL				0.03	0.16			0.36				
	wPt-7426	6BS					0.10	0.28		0.18				
	wPt-1330	7BL									0.45	0.61	0.35	
	wPt-6498	7BL	0.16	0.12	0.02	0.07								
	wPt-9204	NA	0.04	0.04										
Plant height	tPt-5080	1BS	0.07	0.04		-				-				-
	wPt-4419	5A	0.09	0.13		-		0.13		-				-
	wPt-1023	7A		0.06		-		0.12		-				-
	wPt-8981	7BL				-		0.28	0.12	-				-
Spikelets per spike	wPt-0196	1AS				-				-	0.61	0.77	0.55	-
	rPt-7906	1BL				-		0.21	0.22	-				-
	wPt-8168	1BL	0.04	0.05	0.02	-				-	0.38	0.61	0.28	-
	wPt-6012	1BS				-				-	0.61	0.77	0.55	-
	tPt-2163	2AL				-				-	0.61	0.77	0.55	-
	wPt-6509	3A				-				-	0.61	0.77	0.55	-
	wPt-2202	3AL	0.04	0.03		-		0.18	0.22	-	0.61	0.77	0.55	-
	wPt-1151	3BL				-				-	0.61	0.77	0.55	-
	wPt-7572	6A				-				-	0.72	0.75	0.57	-
	tPt-1755	7A				-				-	0.61	0.77	0.55	-
	wPt-1163	7AS				-	0.11	0.46	0.24	-				-
	wPt-4345	7AS				-				-	0.61	0.77	0.55	-
	wPt-4120	7B				-				-	0.40	0.35	0.55	-
	wPt-1330	7BL				-				-	0.61	0.77	0.55	-
Days to maturity	wPt-7426	6BS			-	-	0.14	0.28		-			-	-
Δ^13^C	wPt-0696	6BL			-	-	0.11	0.12		-			-	-
Spike length	wPt-1374	1BS	0.11	-	-	-		-	-	-		-	-	-
	wPt-2395	1BS	0.08	-	-	-		-	-	-		-	-	-
	tPt-6376	3A	0.07	-	-	-	0.12	-	-	-		-	-	-
	wPt-2202	3AL	0.08	-	-	-		-	-	-	0.27	-	-	-
	wPt-4660	4AL		-	-	-	0.25	-	-	-		-	-	-
	wPt-1330	7BL		-	-	-		-	-	-	0.27	-	-	-
			Grain quality traits											
Protein content	wPt-0655	1BS		0.11	-	-		0.08	-	-			-	-
	wPt-5432	3BS			-	-	0.10	0.12	-	-			-	-
Gluten strength	wPt-8770	1A	0.08	0.09	-	-			-	-		0.35	-	-
	wPt-9757	1A			-	-	0.28	0.14	-	-			-	-
	wPt-6274	1AL			-	-			-	-	0.40	0.45	-	-
	wPt-7066	1BL	0.04	0.08	-	-			-	-		0.27	-	-
	wPt-7138	1BS	0.07	0.16	-	-			-	-		0.40	-	-
	wPt-8267	1BS	0.08	0.14	-	-			-	-			-	-
	wPt-7026	2AS	0.03	0.07	-	-			-	-			-	-
	wPt-0398	3AL	0.04	0.06	-	-			-	-			-	-
	wPt-0990	3BL	0.08	0.12	-	-	0.21	0.17	-	-			-	-
	wPt-4412	3BL	0.06	0.07	-	-			-	-	0.40	0.45	-	-
	wPt-6000	3BL	0.05	0.07	-	-	0.21	0.16	-	-			-	-
	wPt-5432	3BS	0.03	0.06	-	-			-	-			-	-
	wPt-1007	4AL	0.03	0.06	-	-			-	-		0.41	-	-
	wPt-3449	4AL	0.13	0.15	-	-			-	-			-	-
	wPt-3506	4AL	0.20	0.21	-	-			-	-			-	-
	wPt-1733	5BL	0.11	0.11	-	-			-	-			-	-
	wPt-8124	6AL	0.06	0.10	-	-			-	-			-	-
	wPt-1121	6B		0.04	-	-	0.32	0.17	-	-			-	-
	wPt-3116	6BS	0.03	0.07	-	-			-	-	0.40	0.45	-	-
	wPt-6916	6BS	0.04	0.13	-	-			-	-			-	-
	wPt-7343	6BS	0.11	0.21	-	-			-	-			-	-
	rPt-7987	7A	0.05	0.07	-	-			-	-		0.35	-	-
	wPt-0321	7A	0.03	0.02	-	-		0.15	-	-			-	-
	wPt-7763	7A	0.29	0.33	-	-	0.11		-	-		0.52	-	-
	wPt-3058	7BL	0.05	0.05	-	-			-	-			-	-
	wPt-9501	NA	0.04	0.07	-	-			-	-			-	-
Yellow Index	wPt-0665	3BL	0.17	0.14	-	-			-	-			-	-
	wPt-6869	7BL	0.04	0.04	-	-			-	-			-	-
Thousand kernel weight	wPt-4835	7A	0.04	0.03	-	-			-	-			-	-
	wPt-0884	7BL	0.20	0.06	-	-			-	-			-	-
Test weight	wPt-7779	2BS	0.03	0.10	-	-			-	-			-	-
	tPt-3599	3BL	0.06	0.07	-	-			-	-			-	-
	tPt-6495	5AL	0.09	0.04	-	-			-	-			-	-

N north, C centre and S south. All experiments were conducted in 2007 except in the south in 2008 as indicated (S08). Dash (-) indicates traits not evaluated at the specific environment.

For quality traits, two markers showed significant association with **PC**, wPt-0655(1BS) in *durum* and *turgidum* in one environment, and wPt-5432 (3BS) in *turgidum* at the two environments. Both markers were monomorphic in *dicoccon* (S5 Table). Twenty-four of the 26 markers that showed significant association with **GS** were identified in the subsp. *durum*. Eleven markers coincided in two subspecies and one, wPt-7763(7A), in the three subspecies. Most of them were located on chromosome groups 1, 3, 6 and 7, and on chromosome 4A. In the subsp. *durum*, the most important markers, in addition to wPt-7763(7A), were wPt-3506 and wPt-3449, both on chromosomes 4AL, wPt-7138 and wPt-8267 on chromosome 1BS, and wPt-7343 on 6BS (*R*^*2*^ from 7 to 21). Some of these markers were monomorphic in landraces of subsp. *dicoccon* and *turgidum* (S5 Table).

Two markers showed significant association with **YI** in the subsp. *durum* at the two environments. The marker located on chromosome 3BL explained 14–17% of the variation for this trait. Two markers from group 7 of chromosomes showed significant association with **TKW** in the subsp. *durum* at both environments. Marker wPt-4835 (7A) was monomorphic in *dicoccon* (S5 Table). For **TW,** three markers on different chromosomes were significant at two environments in the subsp. *durum*.

Overall, the highest number of MTAs was found on chromosome 1B (10 MTAs) mainly for *durum* and *dicoccon*, followed by 7B (9 MTAs) especially in *dicoccon*, and 3B (8 MTAs) mainly for *durum* and *turgidum*, with no MTA on the chromosome 4B. A total of 36 MTAs (33 markers) were on the B-genome, while 28 (24 markers) were identified on the A-genome. The number of MTAs on the B genome exceeded that on the A genome in subsp. *durum*, equalled in *turgidum* and was below in *dicoccon*.

We complemented the association results comparing with respect to the loci monomorphic in each subspecies (S5 Table). For each MTA, we analysed whether the relation bi-allelic marker variation *vs* trait values was in agreement with the phenotypic value and the allelic variant fixed in the subspecies in which the marker was monomorphic. This approach allowed to confirm the following MTAs for the traits: **DH** for wPt-9204, wPt-6498 (7BL), wPt-0489 (2BL), wPt-1890 (6BL) (fixed in *dicoccon*), number of **SS** for wPt-1330 (7BL) and wPt-4120 (7B) (fixed in *durum* and/or *turgidum*), and for wPt-1163 (7AS) (fixed in *dicoccon*), **DM** for wPt-7426 (6BS) (fixed in *dicoccon* and *durum*), **SL** for wPt-1330 (7BL) (fixed in *durum* and *turgidum*), **PC** for wPt-5432 (3BS (fixed in *dicoccon*), and **GS** for wPt-0321 (7A), wPt-6916 (6BS), wPt-9501, wPt-1733 (5BL), wPt-3058 (7BL), wPt-7026 (2AS) and wPt-3449 (4AL) (fixed in *dicoccon*), and for wPt-6274 (1AL) (fixed in *durum*).

## Discussion

We have carried out a genetic diversity analysis of a collection of *Triticum turgidum* L. constituted by a large set of landraces of subsp. *durum* and by a number of *turgidum* and *dicoccon* landraces selected to represent the subspecies diversity in the collection [16]. The broad variation found for all the evaluated traits underlined the large genetic variability of the collection. Using that structured collection, we conducted a maker-trait association analysis for six qualitative and six quantitative agromorphological traits, and six grain quality parameters on the three subspecies, some of them evaluated under different environments.

### Genetic diversity and linkage disequilibrium

Linkage disequilibrium analysis was performed for the whole collection on the basis of the pairwise combinations of 329 DArTs. Previous studies highlighted that LD in collections of elite wheat accessions extends from 1 cM up to 10 cM [5, 38, 39]. In another elite durum wheat germplasm collections the LD value observed was higher and related to a significant germplasm stratification within the collection and a relatively small number of accessions in the subpopulations [8, 40]. Our reference collection, could provide a level of genetic resolution sufficiently high to identify the gene/QTLs affecting the traits of interest and to validate in the future the role of candidate genes.

The genetic variability differed quantitatively among the three durum wheat subspecies. *H* values were 0.36 and 0.32, for the whole collection and for the landraces of *durum* respectively, with the subsp. *durum* representing an 88% of the whole genome. Such results are comparable to the reported by Laidó et al. [8] in a tetraploid wheat collection which included accessions from subsp. *durum*, *turanicum*, *polonicum*, *turgidum*, *dicoccon* and *dicoccoides*. The genotyping of the whole collection and the *durum* subgroup gave an *H* value of 0.42 and 0.39 respectively, with a 94% of the whole collection represented by subsp. *durum*. Taken into account that our collection included only landraces from three subspecies and none wild species, we can consider that the diversity analysed in the present study was relatively high. The level of diversity among the chromosomes was heterogeneous in the three subspecies and complementary in some cases (e.g. Chromosomes 4B and group 2 in the subsp. *turgidum* and *durum*). Genome A showed higher variability than genome B in *turgidum* and *dicoccon*, while similar values for both genomes were obtained in the *durum*. These results not only confirm the genetic differences among the landraces of the three subspecies detected in a previous study [16], but also demonstrated the potential contribution of each of them to the diversity of the whole collection. Laidó et al. [8] also reported similar variability values for genomes A and B in the subsp. *durum*. In contrast, they found that chromosome 1A showed a relatively strong diversity reduction in *durum* (which included modern cultivars), probably due to the selection pressure for obtaining modern varieties with improved quality features. The higher diversity detected in our collection for chromosome 1A in comparison with that found in modern varieties could be a valuable source of genetic variation for wheat improvement since some important characteristics, such as quality traits, are controlled by genes mapped in this chromosome e. g. [41, 42]. For the tetraploid wheat subspecies different from subsp. *durum*, Laidò et al. [8] found that variability of genome A exceeded that of genome B, which is in agreement with ours results. The differences found between A and B genomes reflected the different phylogenetic origin of both genomes.

We identified 356 markers monomorphic in at least one subspecies (S5 Table). These markers were spread across all chromosomes, with a higher percentage on genome B than on genome A, mainly for subsp. *durum*. The analysis of fixed markers in the three subspecies for their homology with *Brachypodium* sequences gave a positive match in 96 markers. The gene ontology of the sequences revealed a wide range of molecular putative functions as transcription factor, transporters, protein kinases, phosphorylases, helicases, etc…being remarkable a drought induced protein in chromosome 3A and a protein associated to disease resistance on chromosome 6B related to two fixed markers in the subsp. *dicoccon*. DArT sequences obtained for 147 fixed markers were searched in databases for homology to wheat sequences. Gene coding sequences were retrieved for 21 markers. These included a Chalcone synthase (*CHS*) gene in 2A, a DELLA protein (*Rht-A* gene) on 6B, a benzothiadiazole-induced MAP kinase 2 (BIMK2) on 6B, and a *SGT1* gene on 3B (both related to disease resistance) and a gene related to biotic and abiotic stress on 3B. Overall, 67 out of the 356 fixed loci matched with the 211 outlier loci found under positive selection across seven tetraploid wheat subspecies [43]. These genes might be selected as having an important role in plant responses to biotic and abiotic stresses, or are in genetic linkage with the loci subjected to selection during the domestication process. Some of these loci fixed were clustered into specific regions on chromosome arms 2BL, 3BS and 4AL, together with several genes/QTLs involved in the domestication of tetraploid wheat, such as the tenacious glumes (*Tg*) and brittle rachis (*Br*) (reviewed in [44]). So, wPt-1349 and tPt-3136 fixed in *turgidum* and *dicoccon* were on the same chromosomes, 3BS and 2AL, to where the gene *Br* [45, 46]. The wPt-7004 (2BL) (fixed in dicoccon) and wPt-1091 (4AL) (fixed in *dicoccon* and *turgidum*) mapped on chromosome 2BL and 4AL, respectively [33], were linked to the *Tg* QTLs that affect threshability according to Peleg et al. [46]. Altogether, the chromosomes with more markers fixed were 7B and 1B in subsp. *durum*, 2B and 6B in subsp. *turgidum*, and 1B, 2B, 3B and group 6 in subsp. *dicoccon*, which is in agreement with the identification of several genes/QTLs subjected to selection during the domestication process in all these chromosomes [43]. Overall, 51 monomorphic loci coincided in *turgidum* and dicoccon landraces. The high number of fixed alleles shared by the two subspecies was in agreement with the higher genetic similarity of subsp. *turgidum* with *dicoccon* than with subsp. *durum* obtained by Laidò et al. [43]. This could be due to taxonomic relatedness between both subspecies, but also to adaptation to similar eco-geographic conditions. So, both subspecies might share similar genomic regions involved in genetic control of important adaptive pathways. In fact, 12 out of these loci were on chromosome 2B, where important morpho-physiological and yield traits have been located [43, 47].

### Phenology influence

In genome-wide association studies, there are two ways to account for confounding phenology influence: (1) statistically accounting for population structure effects and (2) carefully selecting the association mapping panel to reduce the range of phenology [48]. In the present study, the incomplete information of seasonal growth habit was compensated with the high detailed study of the population structure in the whole collection following Wang et al. [49] proposal. The genetic structure of this collection was assessed in a previous study [16]. This analysis indicated that the landraces were separated into nine populations (Pop), with the three subsp. *dicoccon*, *turgidum*, and *durum* largely determining the clustering of them. Significant differences in precocity were identified among the three subspecies, most *durum* landraces had spring habit while most *dicoccon* had winter habit [15], and among some populations within each subspecies [16]. In the subsp. *durum*, the highest differences in phenology were detected between landraces in Pop 2, distinguished by their late heading and high altitude of collection sites, and those in Pop 3, characterized by their earliness and relationship with ancestral lines from North Africa. The association analyses conducted in the current study considered phenological differences between landraces as the genetic structure of the collection was included in the Tassel model and the subspecies separation in the regression analyses.

Besides the growth habit, response to photoperiod is an essential factor for durum wheat phenology [50]. In the present study, some markers mapped close to the *Ppd* (photoperiod response) genes were included in the analyses as wPt-4533 and wPt-7026 linked to *Ppd-A1* on chromosome 2AS, and wPt-6199, wPt-5513 and wPt-7320 linked to the *Ppd-B1* on the 2BS [8, 51]. In the present work, no MTA for adaptive traits were identified for these markers (Table 5). Remarkably, for some of them, one of the allele status was fixed (or markedly more frequent) in one of the three subspecies. In the subsp. *durum*, clear allele differences were identified between the earliest landraces of Pop 3 and the late heading of Pop 2 for wPt-6199 and wPt-7320, close to *Ppd-B1*. These results demonstrated that the highest differences in phenology in our collection were among subspecies, and among the populations defined in the genetic structure analysis of the collection [16]. Both subspecies and populations had a differential eco-geographic distribution supporting that the variation for heading time, photoperiod response and vernalization requirements in wheat landraces were explained by a geographical difference in origin since they are primary mechanisms driving local adaptation [1, 52, 53].

### Association analysis

Meteorological data showed that the crop was subjected to contrasting climatic conditions, which give particular relevance to the associations found across several environments. Overall, 14 MTAs were confirmed for the qualitative traits, most of them in the subsp. *durum* (Table 4). For the quantitative ones, 71 MTAs (for 63 DArTs) were confirmed; 45, 23 and 27 in subsp. *durum*, *turgidum* and *dicoccon*, respectively (Table 5). The highest number of MTAs identified in the landraces of *durum* was in agreement with the high stability between environments showed by this subspecies. Flint-Garcia et al. [11] defined a QTL characterized by a 10% *R*^*2*^ as a ‘major QTL’ when detected in association mapping analysis. Accordingly, ‘major QTL’ were detected in *durum* for all the characters except for DM, number of SS and Δ^13^C. It was also evidenced the high contribution of chromosome 1B of subsp. *durum* to the whole collection variability (89%, Table 3), with relevant MTAs for glume and awn colour, SD, PH, number of SS, PC, SL and GS. Interestingly, the number of MTAs on the two genomes differed among the subspecies, being higher on the B genome in the subsp. *durum* and lower in *dicoccon*. In wild emmer, Peng et al. [54] found that QTL effects and domestication syndrome factors on the A genome significantly exceeded that on the B genome, and that A genome has played a much more important role than the B genome in wheat domestication process. This genetic differentiation of structure and function among genomes between *durum* and *dicoccon* could have occurred during domestication. Remarkably, a high number of MTAs detected in the *durum* landraces were monomorphic in *dicoccon*, mainly for DH, PH, PC and GS.

Only three MTAs matched in the three subspecies; for GC, number of SS and GS (Tables 4 and 5). Overall, 15 MTAs coincided between *durum* and *turgidum*, 14 between *durum* and *dicoccon*, and 4 between *turgidum* and *dicoccon*. Most of these MTAs were for GS, which reflected the stability of this trait across subspecies and environments. The chromosomes with the highest number of coincidental MTAs between *durum* and *dicoccon* landraces were the 1B and the group 3, in agreement with these chromosomes having been implicated in the domestication of tetraploid wheat [43, 54]. The different chromosome location among subspecies for some quantitatively inherited traits could be explained by the different levels of recombination in the three subspecies, the effects of the bottlenecks of domestication, and natural and human selection.

A total of 105 environment-specific associations were identified with the GLM (Q model), of which 60 were confirmed by regression analyses in at least one subspecies. Laidò et al. [8] with the more restrictive model MLM identified 221 MTAs for DH, PH, PC and TKW in a tetraploid wheat collection, and 191 MTAs in a *durum* subset, evaluated over one season and location. These values exceeded ours, indicating that our method was quite restrictive. Also, the genetic structure of our collection, included in the GLM model, considered nine subgroups whereas only two subgroups were included by these authors, despite wild species were also analysed in their study.

#### Marker-trait associations with qualitative agromorphological traits

For **PG**, two highly correlated (*r* = 1) MTAs on chromosomes 3 were shared by *durum* and *dicoccon* landraces. In the subsp. *turgidum*, the association was confirmed at *P*< 0.05 (χ^2^ = 7.66). Laidò et al. [8] in a tetraploid wheat collection found that these two markers were under selection. So, these MTAs were probably related to adaptive mechanisms, resulting in a more erect habit of the *durum* landraces.

Two of the MTAs detected for **SD** in subsp. *durum* and *turgidum* were located on chromosome 5AL (Table 4) in agreement with Kosuge et al. [55]. The wPt-6825(5AL) was mapped [33] close to the *Q* gene according to the QTL identified by Peng et al. [44]. This is a major domestication gene conferring spike shape and threshability in wheat and which has a pleiotropic effect in spike weight, kernel number/plant, kernel number/spike, kernel number/ spikelet, and yield (reviewed in [44]). In the current study, marker wPt-6825(5AL) was monomorphic in the *turgidum* landraces, spikes being significantly less dense. Marker wPt-9094 (5AL) is close to the *PBF-A* endosperm transcription factor gene, which is related to spike development [35].

The location of the MTAs for **GH** on chromosome 1AS, and **awn and glume colour** on 1BS was in agreement with the reported in previous studies [56, 57]. Moreover, the marker wPt-4676(1AS) has recently been mapped very close to *glume hairiness* (*Hg*) gene [58]. The location of these markers near the *Glu-3/Gli-1* loci concurred with previous studies (e.g. [59]). The *Glu-3/Gli-1* loci codified endosperm proteins related to gluten strength [41, 60], which agreed with the coincidence of some of these markers as MTAs for GS in this study. According to the hairless spikes of *dicoccon*, the marker associated with GH was monomorphic in this subspecies.

In the subsp. *durum*, two MTAs for **SC** were identified on 3BL as previously reported [61]. In contrast, the two MTAs identified in *turgidum* were located on the group 6 of chromosomes. To our knowledge, QTLs for SC on group 6 have not been reported previously in wheat. For all these MTAs, the allele status agreed with the fixation of the markers in the *dicoccon* landraces, all of them with red kernels (Table 1). Accordingly, no MTA was found in that subspecies. Remarkably, the wPt-5839(2AS), whose putative related gene is a chalcone synthase (CHS), a key enzyme in the pigments deposition and color of wheat kernels [62], was monomorphic in *dicoccon*. In addition to SC, in the subsp. *durum*, the wPt-0665 (3BL) was associated with **YI** (Table 5). The significant correlation coefficient obtained between both traits in *durum* (*r* = -0.53) indicated that white/amber seeds had significantly (*P*<0.01) higher YI values and higher frequency of the allele presence status (87% of genotypes) than red seeds (27% of genotypes). The higher yellow pigment in amber seeds than in red ones could be explained by the strong relationship existing between the colour of the seed coat and the colour of the endosperm of wheat kernels [63]. Consequently, that MTA was no significant for YI within each colour class. The same conclusion was obtained for the other YI association on chromosome 7B. These results agreed with the lowest values and the uniformity for YI between red grain landraces of *dicoccon*. The inheritance of yellow pigment content has been shown to be relatively complex, as many minor QTLs have been found on all of the chromosomes of durum wheat (reviewed in [64]). However, the major QTLs were mapped on telomeric regions of the long arm of chromosomes of group 7, close to genes coding for Psy enzymes that directs the metabolism towards carotenoid synthesis in the endosperm. In our study, the MTA on 3B had a greater effect (Table 5) than that on 7B. Although the latter was located on the distal zone of chromosome 7, we cannot ensure that is close to the *Psy* genes.

#### Association with quantitative agromorphological traits

Considering **DH**, an important QTL was found in the subsp. *durum* for wPt-6498 (7BL) in the four environments (Table 5). This marker showed the allele absence status in the earliest landraces (Pop 3) and in those coming from the Canary Islands, near North Africa. The presence allele status in *dicoccon* and most *turgidum* landraces (76%) was consistent with the higher DH values in these subspecies. It is noteworthy, that the putative gene function related to this marker was heme oxygenase 1, a key enzyme involved in stress responses as drought (reviewed in [65]) that frequently correlate with DH. Bordes et al. [35] also identified that MTA for DH in bread wheat elite breeding populations, which supports the relevance of this MTA in wheat. Interestingly, the wPt-0489 (2BL), also showed association with DH under the three environments in *durum*. This MTA was also reported in a tetraploid wheat collection evaluated under one environment [8]. According to the genomic sequence of this marker, the putative corresponding gene function was anthocyanidin reductase, a type of protein which induces early flowering and improves seed yield in transgenic tobacco [66]. The importance of this MTA was supported by its presence in subsp. *dicoccon*. The MTA wPt-9204-HD, also detected by Laidó et al. [8], was only confirmed in the colder environments of the centre and northern Spain. In the subsp. *turgidum*, four MTAs were identified for DH, all of them confirmed in the two more contrasting environments (South and North or Centre). One of these MTAs was also confirmed in *durum* in the South and other in *dicoccon* in three environments. The two MTAs, wPt-2858 (2AL) and wPt-3509 (5AL), agreed with those reported by Laidò et al. [8]. Marker wPt-3509 is close to *PBF-A* gene [33, 35] a transcription factor of endosperm genes related to germination, which could cause heading delay in some wheat species [67]. In the *dicoccon* landraces, four MTAs for DH were confirmed in the three environments. In spite of the markers were located on different chromosomes, they were significantly correlated (r from -0.84 to 1, *P*<0.01). These loci showed a different allele status in the varieties from the west zone, significantly later than those from the east zone [16], in which the other allele was fixed. Other studies reported wPt-2858 (2A), tPt-2163 (2A) and wPt-7572 (6A) as MTAs for DH in tetraploid wheat [8, 51].

In wheat, it is important to analyse the relationships between DH and other traits, especially adaptive traits, for a better understanding of the detected MTAs [3, 68]. For subsp. *turgidum* and *dicoccon*, some MTAs for DH were also associated with other traits, as the MTA for DM in *turgidum*, and three for the number of SS and one for SL in *dicoccon*. The positive correlation of DH with DM and number of SS detected in our collection and in a previous study [69] supports the influence of DH on these MTAs and that earliness alleles can reduce spikelet number in southern Europe [70]. In *dicoccon* landraces, the adaptive western-eastern pattern detected in the MTAs for DH was also identified in the MTAs for the number of SS, SL and PG, which confirmed the geographic substructure east–west previously identified in that subspecies [16]. That geographic distribution was related to climatic differences between both zones in temperature, precipitation and ETP. The interchromosomal associations detected in the emmer landraces can be explained by the effect of drift (bottleneck effect), but also might be indicative of areas of the genome where epistatic selection and chromosomal mutations might have reduced the effective recombination rate. So, selection could favour different phenotypic architectures and the conservation of haplotype blocks as has been reported for other species [71]. The peculiarity of Spanish landraces of *dicoccon* was highlighted by Oliveira et al. [21], which considered these wheats as relic varieties that have been preserved due to geographic isolation and small-scale farming. In the subsp. *durum*, the MTAs wPt-0884 (7BL) for TKW, and tPt-3599 (3BL) and tPt-6495 (5AL) for TW were no significant for the late heading *durum* landraces (Pop 2), indicating an influence of DH on these associations.

For **PH**, no MTA was identified in *dicoccon*, in which PH showed the lowest mean value and variation (Table 2). In agreement with our study, QTLs on chromosomes 1BS, 5A, 7A, and 7B have been described in durum wheat [6, 54, 72]. The MTA (wPt-1023) detected in *durum* and *turgidum* was also reported by Laidò et al. [8]. It is noteworthy that the sequence of the marker wPt-7343(6BS) matched with the *Rht-A1* wheat gene inducing dwarfism, and it was monomorphic in *turgidum*, the tallest landraces.

Two important MTAs were confirmed for the number of **SS**, wPt-8168 (1BL) in *durum* and *dicoccon* in the three environments, and wPt-2202 (3AL) in the three subspecies in at least two environments. This result reflects the high stability showed by this trait across environments (S3 Table). The three subspecies showed significant differences for this trait, *durum* having the lowest values and *dicoccon* the highest. Other studies have reported QTLs on 1B and 3A in wild emmer and wheat [54, 73]. We have detected MTAs on other chromosomes as 2A, 6A, 7A and 7B whose location coincided with the reported in previous studies [47, 73, 74]. Peng et al. [54] showed that some QTLs for domestication traits as number of SS and other main yield components were clustered in a limited interval on chromosome 2AL, in which a gene *Br* (*brittle rachis*) was mapped [46]. In our study, some of the markers significantly associated with SS in *dicoccon* were monomorphic in *durum* or *turgidum* landraces, including the one on 2AL.

Carbon isotope discrimination (**Δ**^**13**^**C**) has been suggested as an indirect selection criterion for grain yield under drought stress in wheat [75, 76]. In the present study, we detected an MTA on chromosome 6BL in the subsp. *turgidum*, which had significantly lower mean values and higher variation than the subsp. *durum*. Other authors also reported an MTA on chromosome 6B [77].

For **SL**, MTAs on chromosome 3A were identified in the three subspecies. According to Marone et al. [33], the MTA on 3AL found in *durum* and *dicoccon* was close to the one identified by Ma et al. [78] in a synthetic hexaploid wheat population derived from *Aegiliops tauschii* and tetraploid wheat. In *dicoccon*, the two markers on 3AL and 7BL were correlated (*r* = -1). An MTA on 7BL in the subsp. *dicoccon* was also reported [47]. The coincidental association of wPt-1330(7BL) with SL and number of SS in *dicoccon* could be due to close linkage and/or pleiotropy of genes, reflected in the high correlation between both spike features in emmer landraces (S4 Table). In the subsp. *durum*, the two markers on 1BS were associated with other spike traits besides SL; wPt-2395 with glume and awn colour, and wPt-1374 with SD. The multi-trait association of wPt-1374 can be explained by the strong association between SL and SD (*r* = 0.75**) in this subspecies. The association of wPt-2395 with SL was caused by the significant higher length of red spikes in comparison to the length of the white and black ones in *durum*. The same conclusion was obtained for the wPt-1374 MTA for SL, which indicated that no MTA on 1B was confirmed in the present study. The location of the SL MTA on chromosome 4A in the subsp. *turgidum* was in agreement with the reported by Borner et al. [79].

#### Association with grain quality traits

For **the grain quality traits**, the two MTAs found for **PC** were monomorphic in *dicoccon* landraces, which showed the highest protein content. The MTA on 3BS confirmed in the subsp. *turgidum* was also identified in tetraploid wheat but not in the subsp. *durum* [8]. QTLs for PC on chromosomes 1B and 3B were detected in bread wheat [80].

Most of the MTAs for **GS** measured by the SDSS test were identified in the subsp. *durum*, although some of them were confirmed in two or three subspecies. In subsp. *durum*, several MTAs on chromosomes 4AL, 5BL, 6BS and 7A explained more than 10% of variation at both environments. Some of them were monomorphic in the subsp. *dicoccon*, which had significantly lower GS mean values than the subsp. *durum*. Altogether in the three subspecies, the highest number of MTAs were located on the chromosomes 6B and 3B, followed by 1A, 1B, 4A and 7A. Previous studies located genes for GS in durum wheat on chromosomes of group 1 and 6 [41, 81] and 3BS and 3BL [82]. QTLs for gluten quality on chromosomes 3AL, group 7 [7, 83] and 2AS [84] were also found in bread wheat. To our knowledge, the strong associations found in the present study for markers on 4AL have not been reported previously in durum wheat. In bread wheat, Li et al. [80] found a QTL on 4AL associated with factors not included in the gluten fraction, which explained a substantial amount of variation for GS. In our study, the wPt-7763 MTA on 7A explained from 29 to 33% of the GS variation in *durum* landraces. Although we could not determine the position of this QTL, Furtado et al. [83] identified a gene on chromosome 7L which seems to encode a sulphur rich protein that could support the crossing linking of proteins in gluten.

No marker showed significant association with **V** in more than one location or subspecies in agreement with the low stability found for this trait. However, the most important MTAs identified in *durum* (wPt-1159) and *turgidum* (wPt-2391), covering 16 and 18% of variability, respectively, were located on chromosomes 3B. Other studies have also identified QTLs for hardness in bread wheat on 3BL [85] and 3BS [80].

The two MTAs for **TKW** confirmed in the subsp. *durum* were located on the group 7. One of them, wPt-0884 (7BL), was also identified in tetraploid wheat, but not in the subsp. *durum* [8]. In our study, this marker explained the 20% of the variation in the environment with the better values. However, this association was no confirmed in the late heading *durum* landraces. Similarly, Maccaferri et al. [6] identified one MTA on 7AL and 7BL in durum wheat. MTAs on chromosomes 7 have been also found in bread wheat [68, 86] and in a mapping population from wild emmer and durum wheat [46, 54]. It is remarkable that the QTLs conferring improved TKW on chromosome 7B have been co-localized with QTLs conferring higher grain yield [46].

The association of tPt-6495 (5AL) and **TW** was also identified by Bordes et al. [35] in bread wheat. In agreement with our results, other studies mapped QTLs on chromosomes 2BS, 3B and 5AL in wheat [6, 87, 88]. None of the associations identified in the present study for TW were significant for the late heading *durum* wheat landraces. These landraces possessed lower TW values according to the negative correlation between this trait and DH [89].

Co-linearity between chromosomes within some homoeologous groups was conserved for several MTAs as for PG (3A and 3B in *durum*), SC (6AS and 6BS in *turgidum*), PH (7A and 7B in *turgidum*), number of SS number (1AS and 1BS, 3A and 3B, and 7A and 7B in *dicoccon*), GS (1A and 1B, 3A and 3B, 6A and 6B, and 7A and 7B in *durum*), and for TKW (7A and 7B in *durum*).

## Conclusions

The genetic characterisation and association studies of different wheat germplasm sets will provide researchers comprehensive information of the tetraploid wheat gene pool and the tools to improve the characterisation and utilisation of this germplasm. Markers associated with genomic regions that control traits of agronomic interest will enhance our understanding of the genetic value of the landrace collections and allow to identify new markers associated with key traits whose variation have been lost through domestication and breeding. The identification of monomorphic regions in the genome of one of the subspecies might be very useful for further functional analyses to identify the loci underlying the phenotypic differences between them. The *turgidum* and *dicoccon* landraces analysed in the present study shared several monomorphic genomic regions involved in genetic control of important adaptive pathways which could reflect an adaptation to similar eco-geographic conditions. Moreover, we identified a western-eastern pattern in *dicoccon* landraces for relevant adaptive traits as phenology and spike related-traits. The genetic and phenotypic differences among subspecies and their contrasting distribution among chromosomes and genomes, reflected the different and complementary contribution of each subspecies to the whole collection diversity and their potential value as sources of genetic variation for durum wheat improvement.

The present study identified 85 MTAs for a number of agromorphological and quality traits across environments and/or subspecies. Some of them explained a high phenotypic variation of the traits. Combining the association mapping with an analysis of the signature of selection, 18 MTAs for some adaptive and quality traits were validated. The coincidence of some of those MTAs with other studies indicates that our approach was successful for the detection of MTAs for the traits investigated. Novel MTAs not previously reported, some of them subspecies specific, have been described and will provide new information about the genetic control of complex traits.

## Supporting Information

S1 Fig Overview of the LD parameter r^2^ of the intrachromosomal pairs in the whole collection. The scatterplots show the distributions of the LD parameter r^2^ according to the genetic distance. The horizontal line indicates the 95% percentile of the distribution of the unlinked r^2^, which gives the critical value of r^2^.
(TIF)

S1 Table Description of the testing environments. Meteorological data refer from November to June.
(DOCX)

S2 Table Sum of squares of the ANOVA for the quantitative agromorphological and the grain quality traits for 183 landraces of the subsp. *durum*, *turgidum* and *dicoccon* evaluated in four environments. (DOCX)

S3 Table Pearson’s correlation coefficients between environments for the quantitative agromorphological and grain quality traits. (DOCX)

S4 Table Significant correlation coefficients (*P*<0.05) between the quantitative agromorphological and grain quality traits within each subspecies and environment. (DOCX)

S5 Table DArT markers monomorphic in the subsp. *durum*, *turgidum* and *dicoccon*, chromosome location and putative function. (XLSX)

S6 Table Summary table of MTAs indicating those monomorphic in some subspecies and those found in previous reports. (XLSX)

S7 Table Durum wheat consensus map from Marone et al. (2012). MTAs identified in the present study are marked in bold.
(XLSX)
