# Supplementary figures and images for: Genetic Diversity and Association Mapping for Agromorphological and Grain Quality Traits of a Structured Collection of Durum Wheat Landraces Including subsp. durum, turgidum and diccocon
Source: PLoS One. 2016 Nov 15;11(11):e0166577. doi: 10.1371/journal.pone.0166577 (PMC5113043; doi:10.1371/journal.pone.0166577)

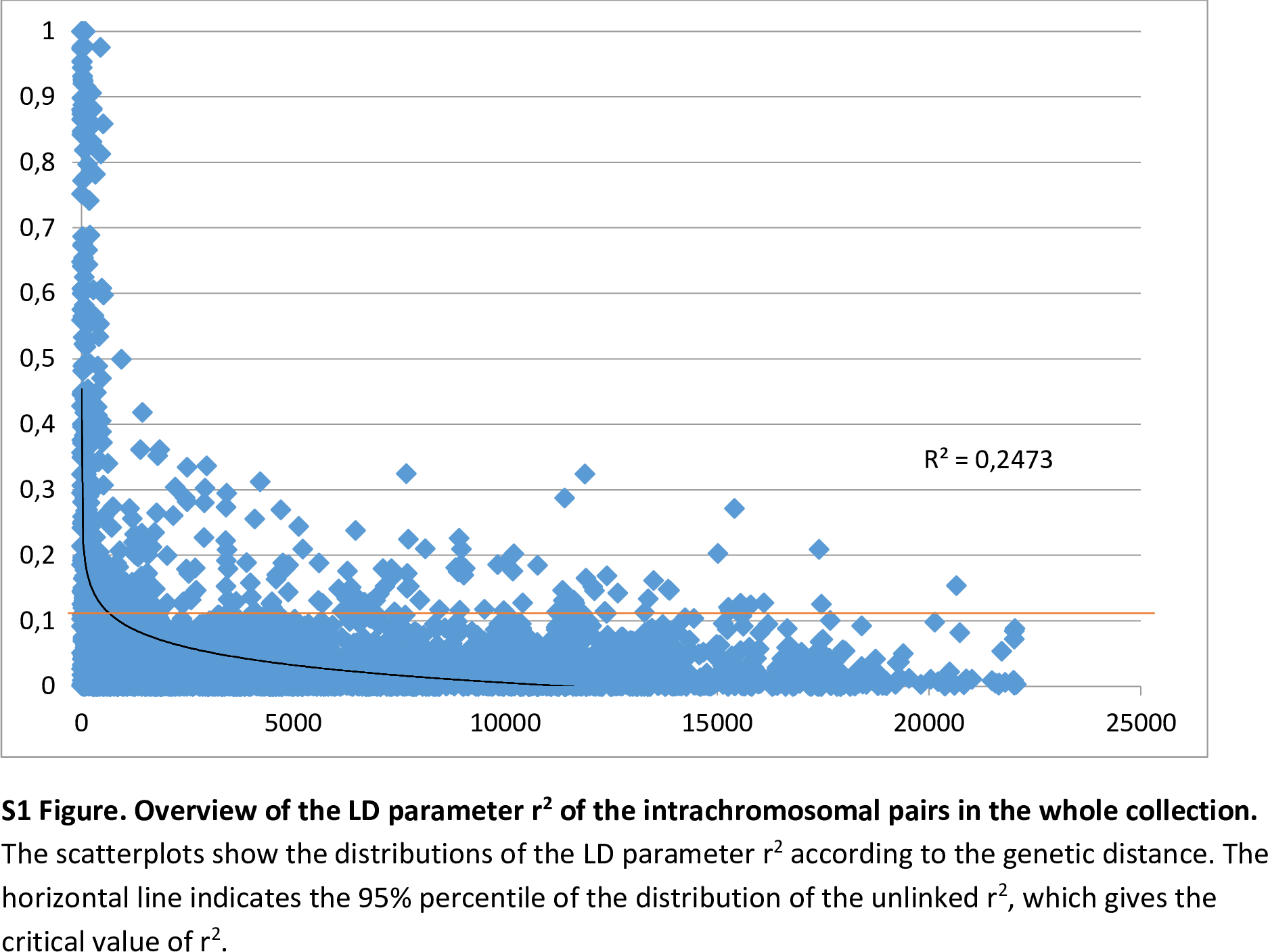

Supplement: S1 Fig — The scatterplots show the distributions of the LD parameter r2 according to the genetic distance. The horizontal line indicates the 95% percentile of the distribution of the unlinked r2, which gives the critical value of r2. (TIF) [file pone.0166577.s001.tif]
