# Supplementary material for: Genetic Diversity and Association Mapping for Agromorphological and Grain Quality Traits of a Structured Collection of Durum Wheat Landraces Including subsp. durum, turgidum and diccocon
Source: PLoS One. 2016 Nov 15;11(11):e0166577. doi: 10.1371/journal.pone.0166577 (PMC5113043; doi:10.1371/journal.pone.0166577)
Supplement: S1 Table — Meteorological data refer from November to June. (DOCX) [file pone.0166577.s002.docx]

**S1 Table**. **Description of the testing environments. Meteorological data refer from November to June.**

| Location | Coordinates | Altitude (m) | Season | Average temperature (°C) | | | Rainfall (mm) |
| --- | --- | --- | --- | --- | --- | --- | --- |
|  |  |  |  | Mean | Maximum | Minimum |  |
| Centre (C) | 40°30’N. 3°17’W | 606 | 2006-07 | 10.2 | 16.3 | 4.2 | 421 |
| North (N) | 41°40’N. 0°20’E | 200 | 2006-07 | 11.4 | 17.3 | 6.2 | 217 |
| South (S) | 36°70’N. 6°20’W | 44 | 2006-07 | 14.1 | 20.8 | 8.5 | 302 |
| South (J08) | 36°70’N. 6°20’W | 44 | 2007-08 | 15.1 | 21.6 | 8.5 | 455 |
