# Supplementary material for: Genetic Diversity and Association Mapping for Agromorphological and Grain Quality Traits of a Structured Collection of Durum Wheat Landraces Including subsp. durum, turgidum and diccocon
Source: PLoS One. 2016 Nov 15;11(11):e0166577. doi: 10.1371/journal.pone.0166577 (PMC5113043; doi:10.1371/journal.pone.0166577)
Supplement: S2 Table — (DOCX) [file pone.0166577.s003.docx]

**S2 Table**. **Sum of squares of the ANOVA for the quantitative agromorphological and the grain quality traits for 183 landraces of the subsp. *durum*, *turgidum* and *dicoccon* evaluated in four environments.**

| Source of variation | Subspecies | Environment | Subspecies x Environment |
| --- | --- | --- | --- |
|  | Quantitative agromorphological traits | | |
| Days to heading | 5024^**^ | 98185^**^ | 389^**^ |
| Plant height (cm) | 11365^**^ | 34964^**^ | 853 |
| Spikelets per spike (number) | 885^**^ | 263^**^ | 6.38 |
| Days to maturity | 554^**^ | 35827^**^ | 40.7 |
| Δ^13^C | 5.22^**^ | 4.55^**^ | 0.59 |
| Spike length (cm)^a^ | 13624^**^ | - | - |
|  | Grain quality traits | | |
| Protein content (%) | 79.1^**^ | 0.05 | 21.8 |
| Gluten strength (mm) | 21.8^*^ | 16.9^*^ | 0.20 |
| Vitreousness (%) | 1612^**^ | 1527^**^ | 3918^**^ |
| Yellow Index | 155^**^ | 77.5^**^ | 1.67 |
| Thousand kernel weight (g) | 1307^**^ | 212^*^ | 760^**^ |
| Test weight (kg/hl) | 41.3^**^ | 660^**^ | 34.5^**^ |

^*^, ^**^ significant at *P*<0.05 and *P*<0.01, respectively

^a^ evaluated at one location
