# Supplementary material for: Genetic Diversity and Association Mapping for Agromorphological and Grain Quality Traits of a Structured Collection of Durum Wheat Landraces Including subsp. durum, turgidum and diccocon
Source: PLoS One. 2016 Nov 15;11(11):e0166577. doi: 10.1371/journal.pone.0166577 (PMC5113043; doi:10.1371/journal.pone.0166577)
Supplement: S3 Table — (DOCX) [file pone.0166577.s004.docx]

**S3 Table. Pearson’s correlation coefficients between environments for the quantitative agromorphological and grain quality traits.**

| Trait | Environment | Environment | Subspecies | | |
| --- | --- | --- | --- | --- | --- |
|  |  |  | *durum* | *turgidum* | *dicoccon* |
| Days to heading | C | N | 0.65^**^ | 0.64^**^ | 0.70^**^ |
|  | C | S | 0.52^**^ | 0.35^*^ | 0.45 |
|  | C | S08 | 0.48^**^ | 0.35^*^ | 0.41 |
|  | N | S | 0.44^**^ | 0.14 | 0.60^*^ |
|  | N | S08 | 0.41^**^ | 0.42^**^ | 0.53 |
|  | S | S08 | 0.45^**^ | 0.18 | 0.45 |
| Plant height | C | N | 0.56^**^ | 0.59^**^ | 0.36 |
|  | C | S | 0.25^**^ | -0.12 | 0.20 |
|  | N | S | 0.23^**^ | 0.23 | 0.26 |
| Spikelets per spike | C | N | 0.63^**^ | 0.41^*^ | 0.95^**^ |
|  | C | S | 0.38^**^ | 0.36^*^ | 0.80^**^ |
|  | N | S | 0.33^**^ | 0.68^**^ | 0.85^**^ |
| Days to maturity | C | N | 0.35^**^ | 0.44^**^ | 0.65^*^ |
| Δ^13^C | C | N | 0.37^**^ | 0.17 | 0.66^*^ |
| Protein content | C | N | 0.23^**^ | 0.28 | 0.39 |
| Gluten strength | C | N | 0.88^**^ | 0.79^**^ | 0.67^*^ |
| Vitreousness | C | N | -0.02 | 0.04 | -0.23 |
| Yellow Index | C | N | 0.79^**^ | 0.71^**^ | 0.37 |
| Thousand kernel weight | C | N | 0.36^**^ | 0.47^**^ | 0.34 |
| Test weight | C | N | 0.27^**^ | 0.08 | 0.29 |

^*^, ^**^ significant at *P*<0.05 and *P*<0.01, respectively
