# Supplementary material for: Genetic Diversity and Association Mapping for Agromorphological and Grain Quality Traits of a Structured Collection of Durum Wheat Landraces Including subsp. durum, turgidum and diccocon
Source: PLoS One. 2016 Nov 15;11(11):e0166577. doi: 10.1371/journal.pone.0166577 (PMC5113043; doi:10.1371/journal.pone.0166577)
Supplement: S4 Table — (DOCX) [file pone.0166577.s005.docx]

**S4 Table**. **Significant correlation coefficients (*P*<0.05) between the quantitative agromorphological and grain quality traits within each subspecies and environment.**

| Trait | Trait | *durum* | | |  | *turgidum* | |  | *dicoccon* | |
| --- | --- | --- | --- | --- | --- | --- | --- | --- | --- | --- |
|  |  | C | N | S |  | C | N |  | C | N |
| Days to heading | Plant height | -0.22^*^ | -0.22^*^ | -0.33^**^ |  |  |  |  |  |  |
| Days to heading | Spikelets per spike^1^ | 0.21^*^ | 0.41^**^ |  |  |  | 0.46^**^ |  |  | 0.76^**^ |
| Days to heading | Days to maturity | 0.44^**^ | 0.75^**^ |  |  |  | 0.81^**^ |  |  | 0.86^**^ |
| Days to heading | Δ^13^C |  | -0.54^**^ |  |  |  | -0.51^**^ |  |  |  |
| Days to heading | Spike length |  |  |  |  |  | -0.36^*^ |  | 0.59^*^ |  |
| Days to heading | Protein content |  | 0.27^**^ |  |  |  | 0.34^*^ |  |  | 0.83^**^ |
| Days to heading | Thousand kernel weight | -0.19^*^ | -0.32^**^ |  |  |  | -0.37^*^ |  |  |  |
| Days to heading | Test weight |  | -0.40^**^ |  |  |  |  |  | 0.59^*^ |  |
| Plant height | Spikelets per spike |  |  | 0.24^**^ |  |  | -0.49^**^ |  |  |  |
| Plant height | Days to maturity |  | -0.23^**^ |  |  |  |  |  |  |  |
| Plant height | Δ^13^C |  |  |  |  |  |  |  |  | 0.66^*^ |
| Plant height | Spike length | 0.20^*^ |  |  |  |  |  |  |  |  |
| Plant height | Gluten strength |  | -0.25^**^ |  |  |  |  |  |  |  |
| Spikelets per spike | Spike length | 0.43^**^ |  |  |  |  |  |  | 0.86^**^ |  |
| Spikelets per spike | Protein content |  |  |  |  | -0.36^*^ |  |  |  | 0.59^*^ |
| Spikelets per spike | Gluten strength | 0.20^*^ |  |  |  | 0.33^*^ |  |  | -0.64^*^ |  |
| Spikelets per spike | Thousand kernel weight | 0.20^*^ |  |  |  |  |  |  |  |  |
| Spikelets per spike | Test weight | 0.34^**^ |  |  |  |  |  |  | 0.77^**^ |  |
| Spikelets per spike | Days to maturity |  | 0.21^*^ |  |  |  | 0.50^**^ |  |  | 0.84^**^ |
| Days to maturity | Δ^13^C | 0.26^**^ | -0.37^**^ |  |  |  |  |  |  |  |
| Days to maturity | Protein content | 0.39^**^ |  |  |  | 0.40^*^ |  |  |  | 0.76^**^ |
| Days to maturity | Vitreousness |  |  |  |  | -0.40^*^ |  |  |  |  |
| Days to maturity | Test weight |  | -0.30^**^ |  |  |  |  |  |  |  |
| Days to maturity | Thousand kernel weight |  |  |  |  |  | -0.43^**^ |  |  |  |
| Δ^13^C | Spike length | -0.27^**^ |  |  |  |  |  |  |  |  |
| Δ^13^C | Protein content |  | -0.45^**^ |  |  |  | -0.50^**^ |  | -0.68^*^ |  |
| Δ^13^C | Vitreousness | 0.24^**^ |  |  |  | -0.37^*^ |  |  |  |  |
| Δ^13^C | Yellow Index | 0.29^**^ |  |  |  |  |  |  |  |  |
| Δ^13^C | Thousand kernel weight | 0.32^**^ | 0.22^*^ |  |  |  |  |  |  |  |
| Spike length | SDSS | -0.20^*^ |  |  |  |  |  |  | -0.57^*^ |  |
| Spike length | Vitreousness | -0.22^*^ |  |  |  |  |  |  | -0.59^*^ |  |
| Spike length | Yellow Index | -0.31^**^ |  |  |  |  |  |  |  |  |
| Spike length | Test weight |  |  |  |  |  |  |  | 0.66^*^ |  |
| Protein content | Vitreousness |  |  |  |  |  |  |  | 0.68^*^ |  |
| Protein content | Yellow Index |  | -0.21^*^ |  |  |  |  |  |  |  |
| Protein content | Thousand kernel weight |  | -0.22^*^ |  |  |  |  |  | -0.60^*^ |  |
| Protein content | Test weight |  | -0.21^*^ |  |  |  |  |  |  |  |
| Gluten strength | Vitreousness | 0.30^**^ |  |  |  |  |  |  |  |  |
| Gluten strength | Yellow Index | 0.25^**^ | 0.27^**^ |  |  |  |  |  | -0.59^*^ |  |
| Gluten strength | Test weight | 0.32^**^ |  |  |  |  |  |  |  |  |
| Vitreousness | Yellow Index | 0.27^**^ |  |  |  |  |  |  |  |  |
| Yellow Index | Test weight |  | -0.22^*^ |  |  |  |  |  |  | -0.80^*^ |
| Yellow Index | Thousand kernel weight |  |  |  |  | -0.32^*^ |  |  |  |  |
| Thousand kernel weight | Test weight |  | 0.35^**^ |  |  |  |  |  |  |  |

^*^, ^**^ significant at *P*<0.05 and *P*<0.01, respectively

^1^Correlations in South were no significant for the subsp. *turgidum* and *dicoccon* except for Days to heading-Spiketes per spike in *dicoccon* (r= 0.61^*^).
